# FungiProteomeDB: a database for the molecular weight and isoelectric points of the fungal proteomes

**DOI:** 10.1093/database/baad004

**Published:** 2023-03-16

**Authors:** Muhammad Rashid, Muhammad Omar, Tapan Kumar Mohanta

**Affiliations:** Department of Data Science, Faculty of Computing, The Islamia University of Bahawalpur, Bahawalpur 63100, Pakistan; Department of Computer Science, Faculty of Computing, The Islamia University of Bahawalpur, Bahawalpur 63100, Pakistan; Department of Data Science, Faculty of Computing, The Islamia University of Bahawalpur, Bahawalpur 63100, Pakistan; Department of Information and Communication Engineering, Yeungnam University, 214-1, Gyeongsan-si 712-749, South Korea; Natural and Medical Sciences Research Center, University of Nizwa, Nizwa, Al-Dakhilya 611, Oman

## Abstract

Proteins’ molecular weight (MW) and isoelectric point (*pI*) are crucial for their subcellular localization and subsequent function. These are also useful in 2D gel electrophoresis, liquid chromatography–mass spectrometry and X-ray protein crystallography. Moreover, visualizations like a virtual 2D proteome map of *pI* vs. MW are worthwhile to discuss the proteome diversity among different species. Although the genome sequence data of the fungi kingdom improved enormously, the proteomic details have been poorly elaborated. Therefore, we have calculated the MW and *pI* of the fungi proteins and reported them in, FungiProteomeDB, an online database (DB) https://vision4research.com/fungidb/. We analyzed the proteome of 685 fungal species that contain 7 127 141 protein sequences. The DB provides an easy-to-use and efficient interface for various search options, summary statistics and virtual 2D proteome map visualizations. The MW and *pI* of a protein can be obtained by searching the name of a protein, a keyword or a list of accession numbers. It also allows querying protein sequences. The DB will be helpful in hypothesis formulation and in various biotechnological applications.

**Database URL** https://vision4research.com/fungidb/

## Introduction

Fungi are one of the most prominent non-vascular, non-photosynthetic heterotrophic organisms on the earth that play an essential role in food, health, medicines and biocontrol application (1–3). The diversity of the fungi ranged from 500 000 to 9.9 million (4). They are the highly abundant organisms on the earth due to their small size and potential to withstand diverse ecological conditions (5, 6). The fungi species have dominated the world from arctic polar to tropical habitats (7–9). Due to their varied genomic and evolutionary plasticity, they are used in various beneficial applications, from enzyme technology to the food industry (10, 11). Some of the fungi are also highly infectious and cause severe diseases in plants and animals (12, 13).

Due to their enormous biological importance, Joint Genome Institute launched a research project named MycoCosm, which targeted sequence 1000 genome (https://mycocosm.jgi.doe.gov/mycocosm/home/1000-fungal-genomes) of the fungi kingdom. Similarly, to date, the fungal genome database (DB) reported 256 genome sequences of the fungi. The National Center for Biotechnology Information (NCBI) also reported 11 940 fungal genome sequences (https://www.ncbi.nlm.nih.gov/genome/browse#!/eukaryotes/). The EnsemblFungi DB (https://fungi.ensembl.org/index.html) has reported the genome sequences of 1506 fungal species. This shows that researchers are trying to sequence more fungal genomes to give us better genomic information about the presence of novel genes and proteins and their biological implications. The presence of a large number of the fungal genome can also help us to understand their evolutionary perspective and adaptation to the diverse ecological niche so that we can use them in various biotechnological applications.

The genome sequence data of fungi have progressed enormously over 5–10 years (14, 15). It allowed us to get the annotated protein sequences of the respective genes (16, 17). The availability of advanced next-generation sequencing technology and powerful proteomics technology, specifically liquid chromatography–mass spectrometry (LC–MS)-enabled high-throughput protein identification and functional assignment tool, led us to identify several novel functional proteins (18, 19). Proteomics has the potential to identify information regarding protein identity, localization and post-translational modification (20, 21). The *in silico* gene model prediction from the genome and its availability of translated protein sequence becomes integral to large-scale ‘omic’ study.

Furthermore, the systems biology approach has enabled us to understand the complex interactions of proteins with other proteins and various other interacting biomolecules (22–24). Enormous efforts are being made for optimal protein extraction from fungi and establishing details of proteomics data relative to their types and abundance (25). Also, considerable efforts are made to catalog proteins from mycelial, secreted or organellar origin across the range of fungi kingdoms (26, 27). Different approaches, including sodium dodecyl sulfate-polyacrylamide gel electrophoresis (SDS-PAGE) or 2D-PAGE fractionation, or the shotgun approach, were used to identify specific proteins using tandem LC–MS to generate the protein data (28, 29). In a study using the 2-DE gel electrophoresis approach, the effect of carbon source, an antifungal drug and gene deletion was explored at the proteomic level (30, 31). The most important aspect of the fungal proteome research is associated with annotated protein sequences as ‘predicted protein’ or ‘hypothetical protein’, making obtaining pertinent information on proteomics data challenging. Ijaq *et al.* mentioned them as known–unknown proteins (32). However, to have uniformity, it would be better to say them as ‘protein of unknown function’ rather than a hypothetical or predicted protein or protein of known–unknown function.

Because the protein is identified and annotated, it exists and is no longer a hypothetical protein. Assigning suitable functions to these ‘proteins of unknown function’ will be an essential aspect of fungal proteomics. However, it is challenging to deduce the function of all the proteins with unknown functions. However, the basic characteristics of a protein can be elucidated from its molecular weight (MW) and isoelectric point (*pI*). The protein gets separated in the 2D-PAGE based on its MW and *pI*. In the first dimension, proteins get separated on a gel using its isoelectric focusing (IEF) that separates proteins according to its *pI*. In the second dimension, the IEF-separated protein gets shifted to SDS-PAGE that separates the protein according to the MW. Therefore, understanding the MW and *pI* (2D) of fungal protein can be essential to hint at their basic biochemical principle. These biochemical details can be quite useful in predicting the function of a protein. The proteins have diverse molecular mass and *pI*s (33, 34). A protein’s shape, size, solubility, MW and *pI* determine its ability to move across different cellular compartments and potential function (35–40). The *pI* indicates the pH at which the net charge of a protein is zero (33). The dissociation constant (pK_a_) of a polypeptide is determined by the presence of seven charged amino acids; arginine, aspartate, cysteine, glutamate, histidine, tyrosine and lysine (41–43). The N-terminal NH_2_- and C-terminal COOH groups of a protein influence the charge of a polypeptide (44–48). Molecular mass and *pI* have been used to determine the position of a protein sequence in a proteome map. It provides the required information to bioinformaticians and genome scientists seeking to understand the molecular basis, subcellular localization and function of a protein (49, 50). Several attempts have been made to create a DB of experimentally validated proteins (51–56). However, it is difficult to experimentally validate every individual protein’s *pI* and MW in a proteome. Hence, we elucidated the MW and *pI* of 685 fungal proteomes and constructed a DB so that users can use the DB and can get basic information of MW and *pI* of the fungal protein and their putative localization and function. The study included information on 7.127141 million protein sequences in total. The DB provides various searching and browsing options to explore 7.127141 million protein sequences efficiently. The DB (i) has a search engine that allows one to explore a virtual 2D map of the fungi proteome, (ii) species search by species name or any other attribute (i.e. the total number of protein sequences, the total number of acidic *pI* proteins, total number neutral *pI* proteins and total number basic *pI* proteins), (iii) search a specific species by accession number, and/or by protein name, and/or by whole sequence or subsequence, (iv), provide summary statistics of individual species as well as of the whole collection and (v) the interface also provides options to copy, save and print the retrieved information, with the ability to export the information in a variety of file types, including Excel, comma separated (csv) and pdf.

## Materials and methods

### Dataset

Annotated protein sequences of 685 fungi species were downloaded from the NCBI, MycoCosm and Ensemble. The downloaded protein sequence files were used to calculate the MW and *pI* using a protein *pI* calculator (http://isoelectric.org/) (IPC Python) within a Linux-based platform (57). The IPC program provided the individual protein sequences’ MW and *pI*. The results were subsequently processed using Microsoft Excel and Python library Pandas.

### Construction of modules

The FungiProteomeDB has three main modules and hence relevant web pages. Other two pages are the home page and feedback page.

#### Construction of home page

The home page comprises a brief description of the DB https://vision4research.com/fungidb/, updates and news feed about the fungi kingdom. The DB offers a user-friendly interface to search for specific protein information and their summary statistics.

#### Construction of summary page

Summary of whole fungi DB https://vision4research.com/fungidb/ is described under four subsections:

- Proteomes vs. proteins summary
- Proteomes vs. *pI* summary
- *pI* protein types summary
- Proteomes vs. MW summary.

It is a webpage with dynamic content. All the statistics were performed using DB manipulation language SQL on a single but large DB table comprising the data of all the 685 species. This DB design makes SQL-based data analysis more efficient as all the data are in a single denormalized table (in DB terminology). If we add or remove some information from the DB, all the statistics will be updated automatically in real-time, avoiding inconsistent data. At the cost of space, we achieved real-time efficiency in data analysis.

#### Construction of species page

Species module https://vision4research.com/fungidb/ provides the following information:

- List of all species with a count of total proteins, which were further classified into total acidic, basic and neutral proteins. Note that it is a dynamically updated list in case of the addition or removal of some species
- Buttons to open virtual 2D proteome map, open species proteins and download Fasta files.

An important feature of the page is the virtual 2D proteome map. A 2D proteome map is a scatter plot where each point is an ordered pair of *pI*s and MW. The data points of proteins were colored/classified according to *pI* values: (i) if 0.00 ≤ *pI* < 7.00, it is an acidic protein, plot it with red color; (ii) if *pI* = 7.00, it is a neutral protein, plot it with yellow color and (iii) if 7.00 < *pI* ≤ 14.00, it is basic protein, plot it with green color.

#### Construction of proteome page and database design

This is the core module of FungiProteomeDB that contains each species’ detailed proteomic information and many search options. From the point of DB design, an individual DB table for each species was created, and each table had the following attributes for each protein: accession numbers, amino acid sequence, name of proteins, MW and *pI*.

The Proteome page has the following two main search modes/modules:

- ‘Substring search mode’: At the initial loading of the page, it fetches all the records of the selected species. The species number selection can be decreased by search filters/search submodules. The search filters of accession number, protein name and sequence fetch all the records from selected species that contains search keyword anywhere inside its column and within the specified ranges of MW and *pI*. All the search fields are joined with the AND operator in the substring search mode. and 5 explain the substring search mode
- ‘Multi-select search mode’: At the initial loading of the page, it fetches no record of the selected species. It can be increased by search filters/search submodules. The search filters of accession number, protein name and sequence fetch all records from selected species of all multi-select values and within the selected ranges of MW and *pI*. The OR operator joins all the search fields in multi-select search mode. Figure 7 shows details about the multi-select search mode.

Both the substring and multi-select search modes of the proteome module have six further submodules or features. They are (i) search by species, (ii) search by accession number, (iii) search by protein name, (iv) search by sequence, (v) search by MW range and (vi) search by *pI* range.

The ‘search by species’ module allows selecting a specific species using a drop-down list or entering a partial name. Internally, it uses SQL wild card string search option.

The module ‘search proteins by protein name’ or ‘keyword’ provides the user with the ability to search for information on a protein using an accession number, protein domain name, MW or *pI* within a species or by a keyword related to the protein domain name. A user can save or print the information once retrieved from the DB. The interface has a list of species names and a keyword search option. A text box is provided for entering a protein domain name or keyword. Instructions for using the module are also provided. The interface also includes a window for displaying search results for a given species. By default, it will display two effects within the first species on the list for entered keywords. The interface has options for copying or printing the retrieved results.

- ‘Sub-search option on retrieved search results’: The sub-search option queries the table that is formed from retrieved data. Users can use the sub-search option to locate a specific entry or entries in the retrieved results. For example, in searching for a protein by name, a user can search a keyword of a protein domain name from the interface.
- ‘Sorting options on each column’: Users can sort any columns within the retrieved results table by using a mouse click.

In module ‘search proteins by accession number’ after selecting a species (otherwise, first species will be displayed by default), when the user starts entering the accession number, autocomplete functionality helps the user to select the accession number from the selected species. This interface also provides the ability to search the DB using the accession number, protein domain name, MW or *pI* within a specific species by using a single accession number or a list of accession numbers. The module has options that allow the user to copy, print or export the retrieved results as an Excel, pdf or csv file. The user can also save the retrieved information for subsequent use.

In the proteome module (page), to search ‘proteins by protein name’, the user has to select a species (otherwise, first species is by default selected). After selecting species, when the user starts entering a protein domain name, autocomplete functionality helps the user select a protein domain name.

In the module ‘search by protein sequence’, we provide the ability to search the DB using a protein sequence within a species using a list of accession numbers provided by the user. The interface has a list box of species names with a protein domain name or keyword search option. A text box is provided for entering a list of accession numbers, and instructions for using this module are also provided. The interface also includes a window for displaying sequences within a selected species name. By default, it will show two results within the first two accession numbers of the first species in the list.

The construction of the two modules—‘search proteins by protein name’ and ‘accession number’, was completed in two steps:

- ‘Preprocessing of the data’: The protein data were collected from each species and placed in a Microsoft Excel file which was generated through IPC software (57). Accession number, protein name, MW and *pI* of each species were mentioned in it. The Excel files were then converted to csv files to import the data into MySQL.
- ‘Database design’: The DB was implemented in MySQL Server, a DB management system. There were 685 tables in the DB; the names and brief summary of each DB table are described in Table 1. After making the DB tables for all species, they were imported using a command prompt. The DB contains protein sequence tables for each of the 685 fungi species. The DB was designed to save all proteomic data of fungi species efficiently with minimum space and high performance. Table 1 shows the tables’ names, purposes and dependencies.

**Table 1.** Summary of DB table

Table name	Purpose	Dependencies
Fungi_kingdom_summary	Summary of fungi kingdom (the whole DB)	No dependency
Species	Summary of individual species	No dependency
species_1	Proteomic details of species number 1 (*Absidia glauca*)	Table name depends on species table id
683 DB tables between the first and last species	Proteomic details of each specific species	Table names depends on species table id
species_685	Proteomic details of species number 685 (*Zymoseptoria tritici*)	Table name depends on species table id

**Figure 1.**
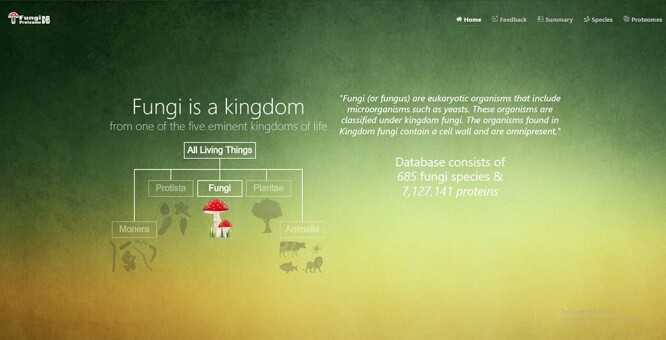
Home page of the FungiProteomeDB. It shows the basic information about the DB.

The construction of the ‘search protein sequence’ was also completed in two steps:

- ‘Preprocessing of the data’: For the development of this module, Fasta files were converted to a csv format using a python language script and then imported into the MySQL Server. The csv files were then compressed for efficient memory use.

In the proteome module (page), to ‘search proteins by molecular weight (MW) range’, the user has to select a species (otherwise, first species is by default selected). After selecting species, the user selects start and end values of MW from a range widget.

In the proteome module (page), to ‘search proteins by isoelectric point (*pI*) range’, the user has to select a species (otherwise, first species is by default selected). After selecting species, the user selects the start and end values of the *pI* from a range widget.

### Front end and backend languages and tools

Languages and tools used in FungiProteomeDB are as follows:

#### Backend languages and tools

The backend uses the MariaDB DB to store all the data. PHP was used as a server-side scripting language that responds to client-side requests and interacts with the DB. An open-source and secure platform Codeigniter 3.1.11 was used to securely run PHP scripts. Summary results were calculated by running SQL queries directly to phpMyAdmin.

#### Front end languages and tools

JavaScript library of JQuery and its different extensions, i.e. JQuery-IU, DataTables and Select2, were used to make website HTML contents attractive and dynamic. Scatter plots (2D proteome map) were created using the JavaScript library convasjs.js. Different CSS libraries of JQuery and Bootstrap were also used to add beauty and attractiveness to web pages.

## Results

In addition to the home page https://vision4research.com/fungidb/, four more web pages were developed (Figure 1).

### Time efficient search interface by species and their attributes

**Figure 2.**
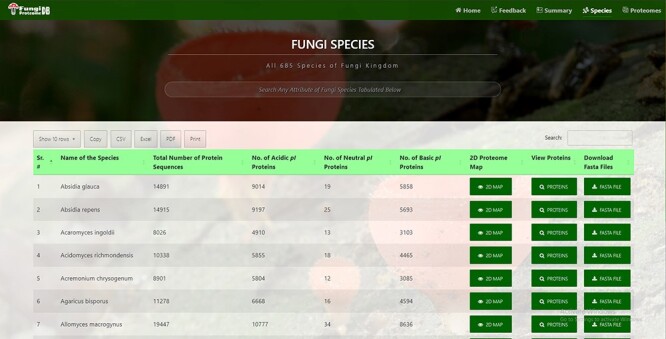
Time efficient search interface by species and their attribute.

In all 685 species names, a total number of proteins were further divided into neutral, acidic and basic *pI* proteins. This information is fetched automatically from the DB table at the time of page load, hence providing the latest information. Species can be quickly searched by name or any attribute available in the table by search box auto-focused and displayed at the top of the page (Figure 2).

#### Species attributes sorting

All the following attributes of species can be sorted in both ascending and descending orders by just clicking the heading of the row:

- Serial or by default order number
- Name of the species
- Total number of protein sequences
- Number of acidic *pI* proteins
- Number of neural *pI* proteins
- Number of basic *pI* proteins.

#### Species records per row

By default, there are 10 rows per page. Users can also change it to 25, 50 and 100 records per page. These customized data can be further used by the users for research with ease as it will provide information in a structured format (suitable for further analysis).

#### Species download in verities of formats

Users can copy the species in a clipboard or download it in csv, Excel, or pdf format. Moreover, a user can print the retrieved results.

#### Species pagination

Users can navigate previous, next or any other specific pages.

#### Virtual 2D proteome map

**Figure 3.**
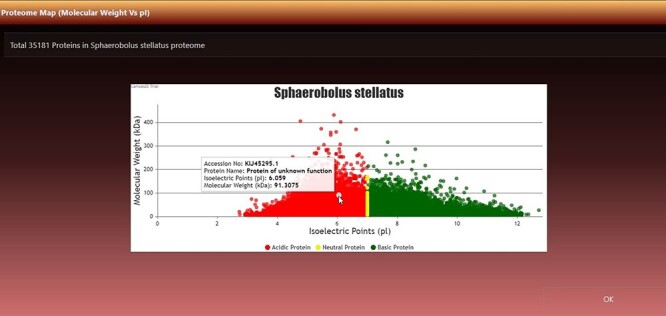
Virtual 2D map of fungal proteome. A representative 2D proteome map of *Sphaerobolus stellatus* is presented here.

It can be viewed by clicking the ‘View Map’ button against a species name (Figure 2, seventh column). Figure 3 shows the 2D map of *Sphaerobolus stellatus*. It is a bi-modal distribution, showing less variation in acidic proteins regarding *pI*, and some values have more MW than neutral or acidic proteins. A few instances of neutral protein can be seen on a vertical line where *pI* = 7. The top left row of the map also shows the total proteins (#dots) represented in the map, and it was 35 181 in the case of *S. stellatus*.

### Proteome search interface

**Figure 4.**
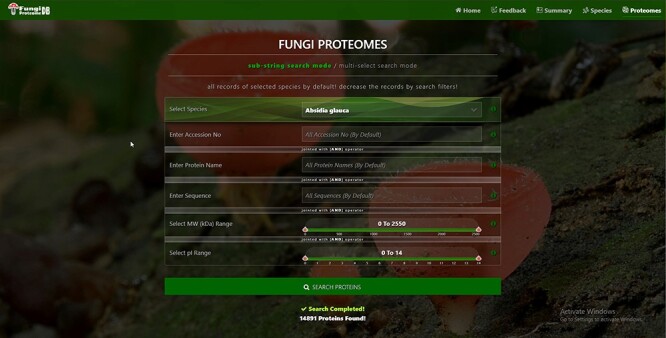
Proteome search interface of substring search mode. User can search the proteome data using any one option or multiple options.

**Figure 5.**
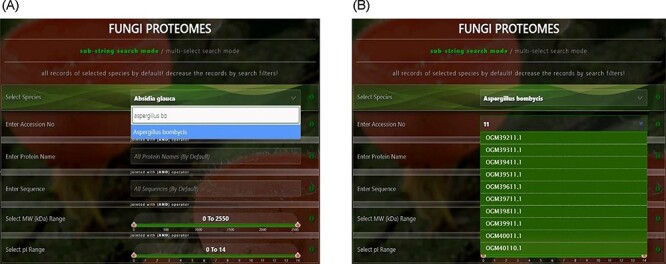
User-friendly interface with autofill option to (A) select species using auto-filling options or from a drop-down list and (B) search specific proteome using accession numbers.

**Figure 6.**
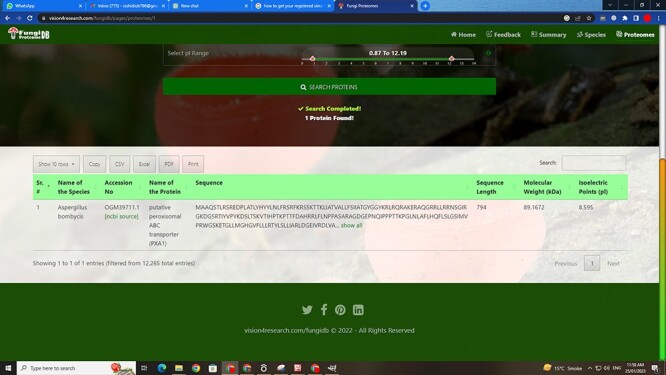
Customized search results using the substring search mode of proteome search interface.

**Figure 7.**
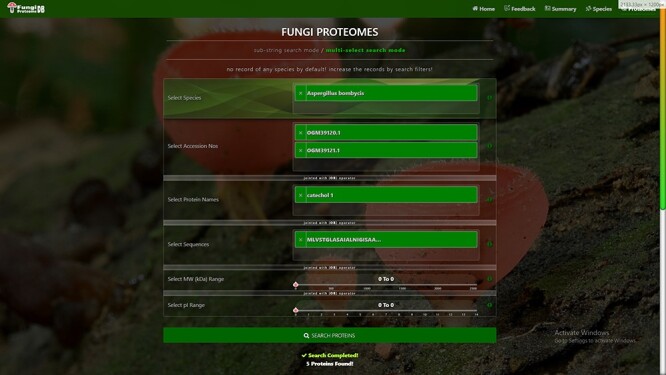
Proteome search interface of multi-select search mode. User can search the proteome data using any one option or multiple options.

**Figure 8.**
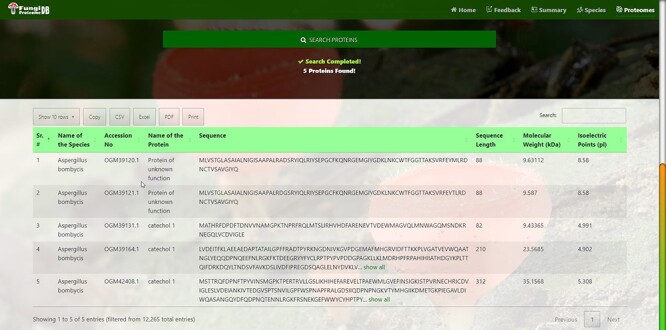
Customized search results using the multi-string search mode of proteome search interface.

There are four types of search options on each search mode in which proteins can be viewed. There are two search modes of (i) substring search and (ii) multi-select search on the proteome page. Figure 4 is the default view of the proteome page https://vision4research.com/fungidb/pages/proteomes when it loads. See Figures 5 and 6 for help with how to use this interface for substring search mode and Figures 7 and 8 for multi-select search mode.

- ‘Proteome/species search’: In both search modes, a single species was selected in this initial search. Figure 5A shows that a user can change species using auto-filling options or from a drop-down list.
- ‘Proteome accession number search’: In substring search mode, by default, all accession numbers of selected species will be selected, but the user can search specific proteome accession numbers (Figure 5B). An autocomplete option is also provided here, in which the accession number will be shown and can be selected. A user can also search substrings on her/his will. Internally, this option of autofill was achieved with wild card search in SQL statement, ‘%substring%’. In multi-select search mode, by default, no accession number will be selected, but the user can select multiple accession numbers by multi-select menu.
- ‘Search by protein name’: In substring search mode, by default, all protein names of selected species will be selected, but the user can also search specific proteins by name. An autocomplete is provided in which the protein domain name will be shown and can be selected. However, the user can also search for substrings if they want. The substring will fetch data in by wild card like ‘%substring%’. In multi-select search mode, by default, no protein name will be selected, but the user can select multiple protein names by multi-select menu.
- ‘Search by sequence’: In substring search mode, by default, all sequences of selected species will be selected, but the user can also search for specific protein sequences. An autocomplete is provided in which protein sequence will be shown and can be selected. However, users can also search for substrings if they want. The substring will fetch data in by wild card like ‘%substring%’. In multi-select search mode, no sequence will be selected by default, but the user can select multiple sequences by the multi-select menu.

Figure 6 also shows the retrieved results table (at the bottom) of the substring search mode and Figure 8 of the multi-select search mode with a search option to apply further search on the retrieved results table. We can see other features of the proteome search interface in Figures 6 and 8 as well:

- ‘Sub-search’: The fetched protein species can further be searched for any attribute shown in the fetched table by search box. All the seven columns (listed below) in the retrieved table can be sorted in both ascending and descending order by just clicking the heading of the row:
- ‘Proteins records per row’: As shown in the first button in Figures 6 and 8, there are 10 rows per page by default. Users can also change it to 10, 25, 50 and 100 and show all records per page.
- ‘Download result in verities of formats’: A user can copy the species in the clipboard or download it in csv, Excel, or pdf format. And by the last ‘Print’ Button, user can print the fetched proteins.
- ‘Pagination of retrieved results’: A user can navigate the previous, next or specific pages.

### Summary statistics

The module provides users with an overview of the overall statistics of the DB. The general statistics provided for the proteome of each species are:

- Sequence count
- Average MW and average *pI* (per each proteome)
- Average MW and average *pI* (per each protein)
- Number of acidic, basic and neutral *pI* proteins
- Percentage of acidic, basic and neutral *pI* proteins.

The overall statistics of the FungiProteomeDB DB https://vision4research.com/fungidb/ is provided in Table 2.

**Table 2.** Statistics of fungi DB

Number of species	685	Number of proteins	7 127 141
Proteomes vs. proteins summary		*pI* protein types summary	
Maximum number of proteins	35 181	Number of acidic (*pI*) proteins	4 407 000
Minimum number of proteins	17	Number of neutral (*pI*) proteins	11 990
Average number of proteins	10 405	Number of basic (*pI*) proteins	2 708 151
Proteomes vs. *pI* summary		Proteomes vs. MW summary	
Maximum *pI* (in all proteomes)	234 364	Maximum MW (all proteomes)	1 210 411 kDa
Minimum *pI* (in all proteomes)	122.1	Minimum MW (all proteomes)	610.27 kDa
Average *pI* (in each proteome)	69 437	Average MW (each proteome)	520 330 kDa
Maximum *pI* (in all proteins)	13.76	Maximum MW (all proteins)	2546.17 kDa
Minimum *pI* (in all proteins)	0	Minimum MW (all proteins)	0.003732 kDa
Average *pI* (in each protein)	6.67	Average MW (each protein)	50 kDa
Proteomes vs. sequence letters summary		Proteins vs. sequences letters summary	
Maximum number of amino acids (all proteomes)	10 940 068	Maximum sequence length (all proteins)	23 089
Minimum number of amino acids (all proteomes)	5417	Minimum sequence length (all proteins)	1
Average number of amino acids (each proteome)	4 693 828	Average sequence length (each protein)	451
Total number of amino acids (all proteins)	3 215 271 966		

## Discussion

Several DBs provide important information about different organisms’ genomic and proteomic aspects. The proteomics DB (https://www.proteomicsdb.org/) reported by Wilhelm *et al.* described the mass spectrometry (MS)-based draft of the human proteome (58). They reported the presence of conserved controlled protein abundance when comparing the messenger ribonucleic acid and protein expression profiles (58). Furthermore, their analysis with integrated drug-sensitivity data enabled them to identify resistant or susceptible proteins for a particular drug (58). The proteome profile can also enable understanding the stoichiometry and composition of the protein complexes (58). ProteomeXchange mission was developed to provide global coordinated standard data submission and dissemination for comparative analysis and extraction of novel findings from the published data (59). PRIDE (http://www.ebi.ac.uk/pride) (PRteomics IDEntifications) DB enables publicly available MS data to publicly accessible data for comparative and functional proteomic. PeptideAtlas (http://www.peptideatlas.org/#) provides access to the compendium of peptides identified in MS experiments (60). It uses the mass spectrometer output files from various organisms and searches using the latest search engines and protein sequences (60). The PeptideAtlas uses MS data of small peptides and enables them to map with the genome of the eukaryotic organism (60). A considerable analytical process with constant statistical validation leads to identifying peptides and proteins (60). The Arabidopsis PeptideAtlas was developed to harness worldwide proteomic data for comprehensive proteomic community resources (61). It provides proteomic information on post-translational modification and splice forms of specific proteins (61). The PeptideAtlas identified 17 858 unique proteins at the highest confidence level (61). The plant proteome DB (http://ppdb.tc.cornell.edu/) reports the experimental data of proteome and MS analysis. PlantMwpIDB reported the proteomic details of plant proteomes using proteins’ MW and *pI* (62). It reports curated information on protein function, subcellular localization and protein properties (63). The fungal secretome DB (https://fsd.snu.ac.kr/) reported the secretary proteins of 158 fungal species comprising 208 883 proteins (64). It comprises 15.21% of the total proteome. Although these fungi-related DBs were constructed to elucidate the proteomic details, they were mainly based on experimental MS data. Therefore, it is challenging to elucidate the proteomic information of a large number of proteins. We used the MW and *pI* data to overcome the issue and construct the DB. This will enable us to find the proteins with acidic and basic *pI* proteins. The basic *pI* proteins usually reside in the basic pH range cellular compartment (65). Proteome-*pI* and Proteome-*pI* 2.0 reported the MW and *pI* of 20 115 proteomes (66, 67). Kozlowski *et al.* reported *pI*s of different proteomes using 21 algorithms (66). They have studied the proteomes of viruses, archaea, bacteria and eukaryotes. However, they have not differentiated the different kingdoms of the eukaryotic lineages. Identifying a specific protein’s MW and *pI* from a specific species is confusing. The lack of a suitable classification of plant, animal and fungi lineage makes it difficult to use effectively. Furthermore, Proteome-*pI* 2.0 does not have a specific option to search the MW and *pI*s of a specific protein in a proteome. A more critical aspect of Proteome-*pI* 2.0 is using 21 different parameters IPC2.peptide.svr19, IPC2.protein.svr19, Wikipedia, Toseland, Turlkill, Solomon, Sillero, Rodwell, ProMoST, Patrickios, Nozaki, Lehninger, IPC_protein, IPC_peptide, IPC2_protein, g Bjellqvist, DTASelect, Dawson, EMBOSS, and Grimsley, IPC2_peptide (66). These 21 parameters result in 21 different MWs and *pI*s for a single protein/peptide. When there are 21 different variations of a single sample, it becomes confusing to accept the suitable output. A particular algorithm is more promising than the 21 algorithms to calculate the MW and *pI*. Therefore, we used only one algorithm in our study, i.e. IPC_protein, and constructed the DB FungiProteomeDB.

## Conclusion and future work

The proposed FungiProteomeDB allows researchers to retrieve information on the MW and *pI* of proteins within the proteomes of 685 fungi species. FungiProteomeDB is a comprehensive DB available for fungi proteomes and contains several modules for searching, retrieving and saving data. Future versions of FungiProteomeDB will make the DB more powerful for obtaining information on the proteome of the entire fungi kingdom. It will also include a protein molecular modeling module to decipher the 3D structure of each protein, target site prediction for metacaspases, palmitoylation, myristiylation and methylation for each protein. This additional information will provide important information to researchers investigating protein modification, function, structure and evolution. Currently (in the proteins search), only one species can be searched by different attributes. In our future version, any species will be searchable at a time by any attribute(s) number. Moreover, we want to add an option for registered users with admin privileges, who can upload new MWs and *pI*s of different species or a protein or its annotation. It will be part of a DB that automatically allows the submission of proteomic data and all the related information. We will also like to search and summarize unique biomarkers in the fungi kingdom [a patch of an amino acid subsequence of length *n* (*n* ≥ 2–5), which is present in the whole proteome file]. Currently, species sort by count values of protein or *pI* is provided; we would also like to add sort by sum values of *pI* or MW.

## Data Availability

All the data used in this manuscript are taken from the publicly available “National Center For Biotechnology Information” (NCBI) database and all the data can be found in our database.
